# Assessing discards in an illegal small-scale fishery using fisher-led reporting

**DOI:** 10.1007/s11160-022-09708-9

**Published:** 2022-03-28

**Authors:** T. Mendo, J. Mendo, J. M. Ransijn, I. Gomez, P. Gil-Kodaka, J. Fernández, R. Delgado, A. Travezaño, R. Arroyo, K. Loza, P. McCann, S. Crowe, E. L. Jones, M. A. James

**Affiliations:** 1 Scottish Oceans Institute, University of St Andrews, East Sands, Fife, KY16 8LB UK; 2 Facultad de Pesquería, Universidad Nacional Agraria La Molina, Av. La Molina s/n, Lima, Peru; 3 IT Services, University of St. Andrews, Walter Bower House, Guardbridge, KY16 0US UK; 4 Biomathematics and Statistics Scotland, Peter Guthrie Tait Road, Edinburgh, UK

**Keywords:** Effort, Tracking, Bycatch; mobile Application, Blue agenda, Trawling

## Abstract

About a third of all marine fish in the world are caught in Small-Scale Fisheries (SSF). SSF are increasingly recognised as essential for food security and livelihoods for vulnerable and economically fragile communities globally. Although individual SSF vessels are usually perceived as having little impact on the ecosystem, the cumulative impact of gear type and number of vessels may be substantial. Bottom trawling is a common fishing method that can greatly influence the marine ecosystem by damaging the seafloor and generating high levels of discards. However, appropriate sampling coverage using on-board observer programmes to collect these data from SSF are rare, as they are expensive and pose logistical constraints. A mobile App was used to assess whether self-reporting by fishers could provide reliable fine-scale information on fishing effort and discards over time in an illegal shrimp trawling fishery in northern Peru. Maps depicting the spatial distribution of trawling effort and the proportion of discards from observers and fishers were compared using the Similarity in Means (SIM) Index, which ranges from 0 when spatial patterns differ completely to 1 when spatial patterns are very similar. High levels of agreement between spatio-temporal patterns of effort (SIM Index = 0.81) and discards (0.96) were found between fisher and observer maps. Moreover, far greater spatial coverage was accomplished by fishers, suggesting that self-reporting via an App represents a useful approach to collect reliable fisheries data as an initial step for effective monitoring and management of these fisheries.

**Supplementary Information:**

The online version contains supplementary material available at 10.1007/s11160-022-09708-9.

## Introduction

Bottom trawling, where fishing gear such as beam, otter trawls or dredges are towed over the seafloor, is the most widespread human source of physical disturbance affecting seabed habitats (Amoroso et al. 2018). Among the main impacts associated with bottom trawling are the alteration of habitat structure, reduction of faunal biomass, productivity and species richness (Collie et al. 2017; Sciberras et al. 2018) which can lead to changes in the trophic structure and function of ecosystems (Thrush and Dayton 2002; Tillin et al. 2006; Pusceddu et al. 2014). In addition, high discard rates (where species or sizes of species that are not targeted are thrown overboard usually dead or dying) are attributed to bottom trawls. It is estimated that bottom trawl fisheries contribute to 45% of all discards (4.2 million tonnes) (Pérez Roda 2019). Discarding practices are controversial resulting in waste, juveniles of other commercial species are overexploited (usually affecting co-occurring fisheries), and biodiversity and protected and endangered species are threatened (Dayton et al. 1995; D’Agrosa et al. 2000; Lewison et al. 2004; Harrington et al. 2005).

Most of what we know about the impacts of bottom trawling come from studies of large scale fisheries. Even though Small-Scale Fisheries (SSF) contribute to about a third to half of all fish caught in the sea (Kelleher et al. 2012; Pauly and Zeller 2016), data on discards are limited (Lewison et al. 2014; Suuronen and Gilman 2020). While in general, it is acknowledged that SSF contribute very little to global discarding (Zeller et al. 2018) and have low discard rates (~ 4%) (Kelleher 2005), it is increasingly recognised that the magnitude of their impact on the ecosystem is more likely related to the quantities of vessels involved in the activity and the types of gear used (Shester and Micheli 2011; Belhabib et al. 2018). Thus, shrimp trawling by SSF can have discard rates comparable to those observed in industrial fisheries (Pérez Roda 2019).

Several methods are used to monitor discards worldwide, including at-sea observer programmes, remote electronic monitoring (REM), logbook or smartphone reporting, fisheries collaborative sampling schemes and interviews (Mangi et al. 2015; Suuronen and Gilman 2020). Independent on-board observer programmes have generally been depicted as the most reliable means to assess discards (Pérez Roda 2019; Suuronen and Gilman 2020). This remains the most widely used method, and in the Food and Agriculture Organisation (FAO) third assessment of global marine fisheries discards, more than 78% of the discard rates were obtained from this source (Pérez Roda 2019). While on-board observers provide accurate and comprehensive information on discards and associated information (e.g. assessment of probability of post-release survival, environmental variables, vessel information) they remain an expensive method to monitor discards (Suuronen and Gilman 2020). In SSF, the large number of vessels, the remote and dispersed nature and vessel size constraints (i.e. sufficient deck space to host an observer) limit appropriate sampling coverage by this means. In recent years, REM which includes on-board cameras, have been used as an alternative method to obtain reliable information on bycatch and discards (Kindt-Larsen et al. 2011; Glemarec et al. 2020). One of the advantages of REM is to provide spatial information, from which main fishing grounds and effort can be estimated. While REM has been proven effective to assess bycatch in SSF (Bartholomew et al. 2018), their wide-scale deployment is hindered by the relatively high price of the equipment compared to SSF revenue, the number of vessels involved and the capacity of authorities to manage and utilise large quantities of image data in an operational context. For SSF, fisher-led reporting on discards may allow a wider, more cost-effective sampling coverage.

The use of logbooks and more recently smartphones has allowed fishers to self-record data on bycatch and discards which is sent to managers in real or near real-time (Merrifield et al. 2019). However, some of the challenges faced by self-reporting are a lack of time, motivation and training by fishers to report accurate data (Lordan et al. 2011; Sampson 2011; Mangi et al. 2015). As discards are considered bad practice, under-reporting may occur, especially if there is an economic or regulatory disincentive to report (Walsh et al. 2002; Hamer et al. 2008).

In Peru, only small-scale fisheries which are not considered to have a high impact on the ecosystem are allowed to operate within 5 miles of the coast (General Fisheries Law 2001, DS-012-2001-PE). Nevertheless, the small-scale shrimp fishery operates illegally within inshore areas in northern Peru with little and ineffectual policing and with high levels of conflict with other small-scale fisheries operating in the area (Mendo et al. 2020). The illegal nature of this fishery has so far prevented management or monitoring of this fishery by regulatory authorities and therefore there is no detailed information on the magnitude of discards that, according to preliminary studies, range between 19 and 95% of the total catch weight (Ordinola et al. 2008; Salazar et al. 2015). Preliminary research suggests that this fishery makes a significant contribution to the local economy and provides more than three times the minimum monthly wage (S./930, ~ US$225 in 2020) for crew members and more than 12 times for vessel owners in this area, suggesting a very strong economic incentive to continue to pursue these illegal activities (Mendo et al. 2020). Fishers in northern Peru have repeatedly requested that the Peruvian Marine Institute (IMARPE) conduct research to reduce the level of impact of this fishery (pers. com. Alex Eche, head of the fisher’s organisation) and find sustainable measures that would allow the formal regulation and legitimisation of the fishery. In this context, fishers contacted members of the National Agrarian University, to identify ways to increase the sustainability of the fishery. The DYNAMICOPERU project was established with three main objectives: (a) assess spatio-temporal variation in bycatch and discard rates to identify areas and periods of high risk to the environment, (b) modify the trawl net to reduce bycatch, and (c) evaluate the potential economic impact of management measures arising from the two previous objectives. Due to the illegality of the fishery, the implementation of a government-led on-board observer programme would be difficult, therefore the feasibility of using a self-reporting low-cost technology (cell phone application) was assessed to monitor the impact of this small-scale shrimp fishery. We hypothesised that, due to the illegality of the activity and hence the strong incentive to under-report, discard rates and fishing activities reported by fishers would be lower than those reported by observers. We specifically assessed data from fishers against data recorded by on-board observers through comparison of (i) the spatial footprint of trawling activities, (ii) the proportion of discards (kg discarded with respect to total catch) reported over time, and (iii) spatio-temporal changes in discards. We then discuss the drivers that can lead to successful self-reporting by fishers.

## Methods

### The shrimp trawl fishery

In Peru, trawling is prohibited within 5NM from the coast (Fishing Law Decree Nº 25,977) but it occurs nevertheless, causing conflicts with other fishers. The small-scale shrimp trawling fleet do not have access to official jetties and distribution channels, a subset of buyers will meet them offshore and collect their catch. Between 49 and 313 tonnes per year of langostino café (“coffee shrimp”) *Penaeus californiensis* were landed by this fleet operating in northern Peru during 2014–2018 (IMARPE 2019). The Fisheries Association (“Asociación de Pescadores Artesanales de la Caleta Constante, Sechura”), which involves about 100 shrimp trawling vessels operating in northern Peru, approached researchers at the national Agrarian University (UNALM) seeking ways to adopt more sustainable trawl fishing practices as a way to legitimise the fishery. This association facilitated access to trawl vessels, fishing information, experiential knowledge and contributed to co-development of recommendations to improve the sustainability of the trawl fishery (Mendo et al. 2020).

**Fig. 1:**
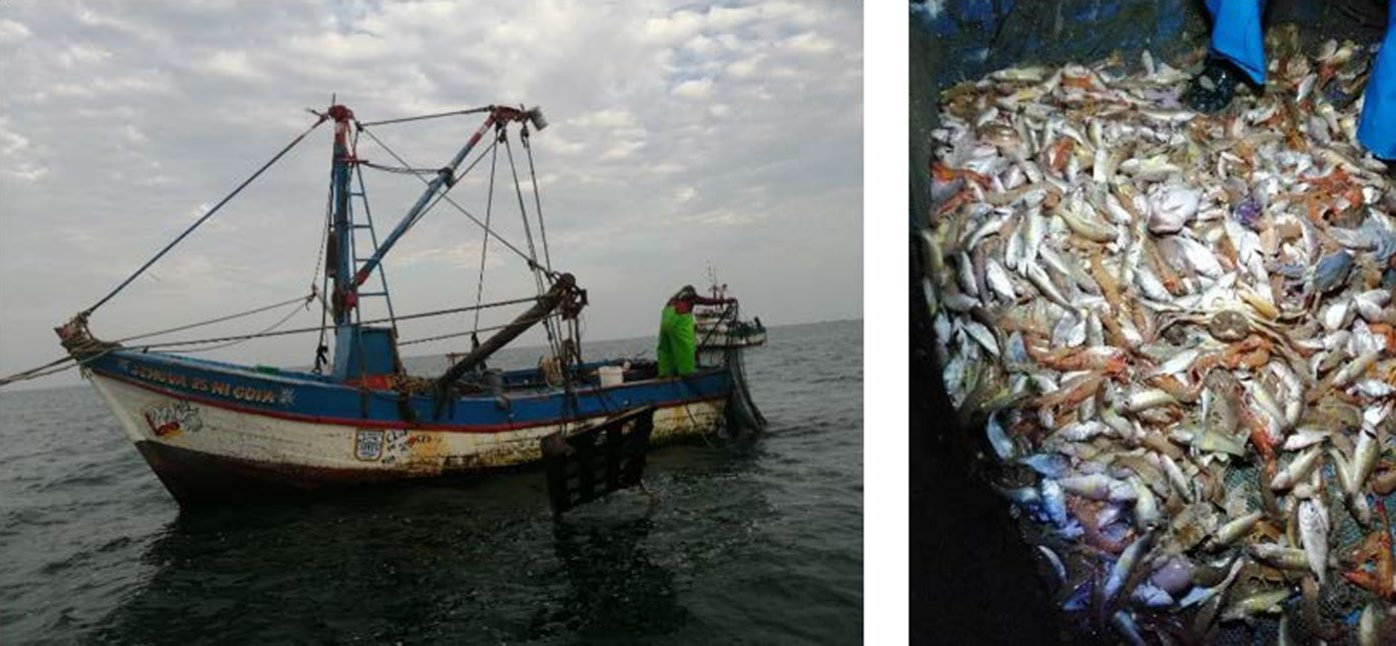
Left: Typical small-scale shrimp trawl vessel in Peru. Doors visible on the side. Right: Example of catch including target species (coffee shrimp) and other commercial species such as sand-perch

This study focuses on trawling vessels operating in the northern Talara province, between 4.4 and 4°S, which target coffee shrimp. There are around 30 vessels operating in this area (Mendo et al. 2020). The fleet is fairly uniform: vessels are < 10 m long, with a storage capacity of 7 tons and an engine power of 120 HP on average (Fig. 1). Usually 1 skipper and 3 crew members operate each vessel. This fishery operates mainly at night using otter trawls with nets 18–24 m in length and 19–25 mm codend mesh size (Mendo et al. 2020). On a typical day, fishers will begin steaming to fishing grounds after sunset, then deploy the net and trawl at 2–3 knots for 2–3 h in each location. Usually 3–5 trawls are conducted per fishing trip. Once on board, the catch (Fig. 1) is separated into retained catch (including species such as coffee shrimp and other commercial species) and the rest (small fish, invertebrates and algae with no commercial value) is thrown overboard as discards.

### Data collection

A total of 12 vessels participated in this part of the project from October 2019 until March 2020. All skippers agreed to host an on-board observer, and 9 skippers agreed to use the App (see below). To avoid an on-board observer effect on reporting, and also because fishers tended to ask observers for the weight of their catch to fill in fields in the App, we compared information submitted by fishers when there was no on-board observer present, and evaluated if fishers’ submissions could be used reliably to monitor the impact of a small-scale shrimp fishery. When an observer was on-board a vessel, we used only the data collected by the observer and data submitted by the fisher via the App were not incorporated in the analyses to avoid inflation of the correlation between the two sources of information.

#### Fishers

The mobile application Pescar App (McCann and Mendo 2019), which runs on devices using the Android operating system was developed openly on GitHub under the terms of the open-source MIT License. It allows fishers to record their location at 3 min intervals from when they turn the tracking feature on until they turn it off; the weight of catch for each haul and the weight of the main commercial species retained for each trip (See Supplementary Material 1). The data was submitted to a server hosted at the University of St Andrews. It was recognised from the outset of the project and through conversations with fishers that the design of the App should minimise the time they would spend self-reporting. Therefore, only six main commercial species were included in the App after a first round of discussions with fishers: coffee shrimp; sand-perch *Diplectrum conception*; flounder *Etropus ectenes*; squid *Lolliguncula sp.*; white shrimp *Litopenaeus vannamei* and; guitarfish *Pseudobatus planiceps*.

As an incentive to participate in the project and submit data regularly, seven fishers were provided with a smartphone and the associated data costs for submitting the information was covered by the project. Phones cost ~ $US120 and data cost $8 per month, and a verbal agreement was made where the cost of data would be continuously supplied (by paying for the SIM card monthly cost) if they were reporting their trips adequately. These fishers were also allowed to keep the phones supplied after the conclusion of the research. Two additional fishers volunteered to use their own cell phones and covered their own data costs to participate in the trial. A narrative of the ethical statements covering the collection, storage and use of the data provided by fishers was included as an opening on-screen introduction to the App which fishers were required to accept before being able to use the App.

Training of fishers was conducted in different stages. First, a one-hour workshop was conducted collectively in July 2019, where the objectives of the project and the App’s user interface were presented. A step-by-step explanation of how to use the App was presented via a Powerpoint presentation, followed by a personalised one to one session where each fisher was shown how to use the App (by providing information for a hypothetical trip) by one of the four observers. In the following three weeks after this workshop, the on-board observers went on board participating fishing vessels to overview the fishers’ submission process and provide feedback and repeat training as needed. During this period, fishers commented on the functionality of the App which helped identify errors in the software which were communicated to the Applications development team. Once all programming issues were resolved and fishers knew how to submit the required fields of information, the data sent to the server was assessed every two weeks to evaluate the number of fishing trips conducted per fisher, if the spatial information provided was sufficient to characterise a trip, and if they provided associated catch data. If we observed any anomalies with respect to the number of trips, or if information was missing, an on-board observer would call the fisher to inquire about the reason for this lack of reporting and to urge them to continue reporting.

As fishers do not have weighing scales on their vessels, the weight of catch for each haul had to be estimated. On-board observers asked fishers to estimate the weight of the catch in 48 trips (with 162 hauls) to assess how well fishers predicted the real weight of the catch. A linear regression between the measured weight by observers and the estimation of weight by fishers showed that fishers were very good at predicting the weight of the catch (see Supplementary Material 2, Intercept = − 1.14, slope = 0.98, R2 = 0.898).

#### On-board observers

Four observers (in teams of two for logistical and safety reasons, allocated randomly to vessels) collected information on-board of 11 of the 12 participating shrimp trawling vessels from October 2019 to March 2020. For each trip positional data were collected every three minutes, using a handheld Garmin Etrex 20. Observers also recorded the name of the vessel, departure time, start time and end time for each haul, and time at which the trip was finished. For each haul, total weight of the catch in the net was recorded on-board using a Kambor digital scale (1 tonne capacity and 0.5 kg precision). Whilst this was not a motion compensated weighing system the sea conditions in which this fishery operates are relatively benign and vessel roll and pitch is quite limited. Crew members sorted the catch and retained commercial species which included coffee shrimp, sand-perch, and flounder. Observers weighed each commercial species weight using a Kambor scale (100 kg capacity and 20 g precision).

The proportion of discards for each trip was estimated as follows:

1 $$Dp \, = \, \left( {Tc \, {-} \, Cc} \right)/Tc$$

where Dp = Discard proportion, Tc = total catch per trip, Cc = Commercial catch per trip.

### Data cleaning and analyses

#### Positional data

Identifying trips from positional data reported by fishers requires a series of steps to deal with possible reporting errors (e.g. fishers turned on the tracking feature without being engaged in a trip). Following the approach of James et al. (2018), first, the latitudes and longitudes were examined to verify that positions were located in the study area. Duplicates and points on land (a 10 m buffer around the coastline was considered) were removed. The temporally ordered sequence of positional records (trajectory) was then created for each fishing trip (a unique trip identifier was assigned for each user-date combination) using the adeHabitatLT package in R (Calenge 2006). The shortest trip duration was 3.5 h (from observer data). Therefore, trajectories shorter than three hours were removed from the analysis. 95% of trips consisted of more than 75 positional records, so a conservative threshold of 50 points was considered as a minimum to characterise a trip. Speeds greater than 6.5 knots were removed, as no vessel operated at higher speeds. Where gaps in positional data exceeded 5 km and there was only one positional record at either side of this gap, this record was removed. A circular spatial buffer zone of 500 m was set around the first and last positional record to avoid incorporating locations with low speeds as a result of transiting anchorage zones or harbours. Trajectories were standardised by regularly sampling linearly interpolated locations every three minutes, as this rate was considered to convey sufficient temporal resolution to identify fishing activities (usually lasting more than one hour).

#### Inferring trawling activities

For trips with on-board observers, overlaying the haul start and end time recorded by observers and the timestamp in the positional data allowed accurate mapping the location of trawling activities. For fishers’ trips, Random Forests (RF, Breiman 2001) were used to infer when vessels were engaged in trawling activities based on positional data. RFs are a machine learning classification technique that combines multiple decision trees for more accurate classification (Cutler et al. 2007). Each tree assigns the most likely class by recursive binary partitioning (tree branch-like structures) that increases the homogeneity within groups based on a range of observations about that item. The distance between observations, relative angle between positions, and the time of the day were used as predictors of trawling activities. The Random Forest model was fitted using the R package randomForest (Liaw and Wiener 2002). We used information from 333 observer trips conducted from June 2019 to March 2020 (which included vessels not participating in the App trial) to assess the performance of the model’s output to observers’ ground-truthed data on trawling activities. Fourteen vessels hosted on-board observers during this period; therefore, we randomly divided the 14 vessels into two sets of seven vessels for training and prediction, respectively, to test for out of sample accuracy of the model. Accuracy was defined as the number of correctly classified instances (for both trawling and not trawling) with respect to their total number of locations. The model predicted trawling activities with a 90% accuracy.

#### Comparing discard proportions over time

Discard proportions reported by observers and fishers via the App were compared over time using a Generalised Linear Square model. Differences in spread in the data provided by fishers and observers in each month were addressed by using the varIdent structure and an autoregressive moving average (ARMA) model was used to deal with temporal autocorrelation in the R package nlme (Pinheiro et al. 2020). Significant differences between months were compared using the R package multcomp (Hothorn et al. 2008).

#### Comparing spatial information

We estimated the spatial distribution of effort (time spent trawling) from spatial data collected by observers and fishers from October 2019 to March 2020. Trawling activities were portrayed in a 500 × 500 m^2^ grid by adding each positional record in each grid cell using the R package raster (Hijmans 2020) and multiplying it times three minutes. The proportion of discards in each trip was assigned to each associated positional record and a weighted mean value (based on the number of points in each grid cell) estimated for each 500 × 500 m^2^ grid cell. This process resulted in a set of maps of trawling effort for observers and fishers, and a set of maps for the proportion of discards from observers and fishers.

To investigate the similarity between the maps for observers and fishers, metrics were used based on the Similarity in Means Index (SIM index, Jones et al. 2016), which provides a measure of similarity in local spatial patterns between two maps (observers and fishers). As the observer and fisher maps were derived from tracking data, the resulting densities have underlying autocorrelation. The SIM index accounts for spatial dependencies between continuous-valued cells, providing an unbiased comparison between the same cells in different maps and retaining locational information about similarities between the underlying maps being compared (Jones et al. 2016). The SIM index ranges from 0 to 1 when 0 denotes dissimilar means in the underlying maps (e.g. the two maps show different local abundances) and 1 denotes similarly high or low values in the underlying maps (e.g. the two maps have similar local abundances). To investigate similarities between fishers and observer maps, a neighbouring spatial unit of 3 × 3 adjacent grid cells was chosen. Due to the greater number of submissions by fishers, data on effort (time spent trawling) was normalised to values between 0 and 1 for both observers and fishers, to allow for comparison in spatial patterns using the SIM index.

## Results

### Data reporting by fishers

Between October 2019 and March 2020, information was available for 277 trips, although not all trips had enough data to identify trip location and discard rates. Sufficient positional data were reported by 9 fishers using the App to identify 243 fishing trips. Individual fishers reported between 8 and 53 trips for this period, with highest numbers reported Oct–Dec (62–65 trips per month), and lowest from Jan–Mar (26–27). It is important to point out that this decrease in the number of trips reported was at least partly because of changes in target species by the fleet (and therefore a change in fishing gear, for which no reporting was expected using the App). Furthermore, an increase in control and surveillance activities by the navy was observed during Jan–Mar (Gomez, I.; pers. obs). As these activities can result in gear and catch confiscation, fishers stop trawling if they expect more patrolling activity in the area. Fishing activities stopped in Mid-March for safety reasons due to the outbreak of COVID-19.

The weight of the total catch was estimated by fishers for 1158 hauls in 240 trips. For most trips, fishers reported between 3 and 6 hauls, which matches the number of hauls usually reported by observers. Shrimp catch was reported for 239 trips, while other commercial species were reported for 212 trips. Of the 243 trips for which positional data were available, 65 trips had no associated catch data. This suggests that fishers recorded the track but either forgot or neglected to provide associated bycatch information in ~ 25% of the trips, as the likelihood of having zero catch per haul is considered very low (based on on-board observers data). Conversely, 37 additional trips provided information on discards, but had no associated positional data. Closer examination of these trips showed that fishers either completely neglected to enable the tracking function in the App (14 trips) or stopped the tracking function, as not enough positional data was collected to identify a trip (23 trips). There were sufficient data to calculate the proportion of discards for 210 trips.

### Data reporting by observers

From October 2019 to March 2020, observers went on board 11 different vessels for a total of 35 fishing trips (3–9 trips per month. 173 hauls in total).

### Spatial footprint of trawling activities

**Fig. 2:**
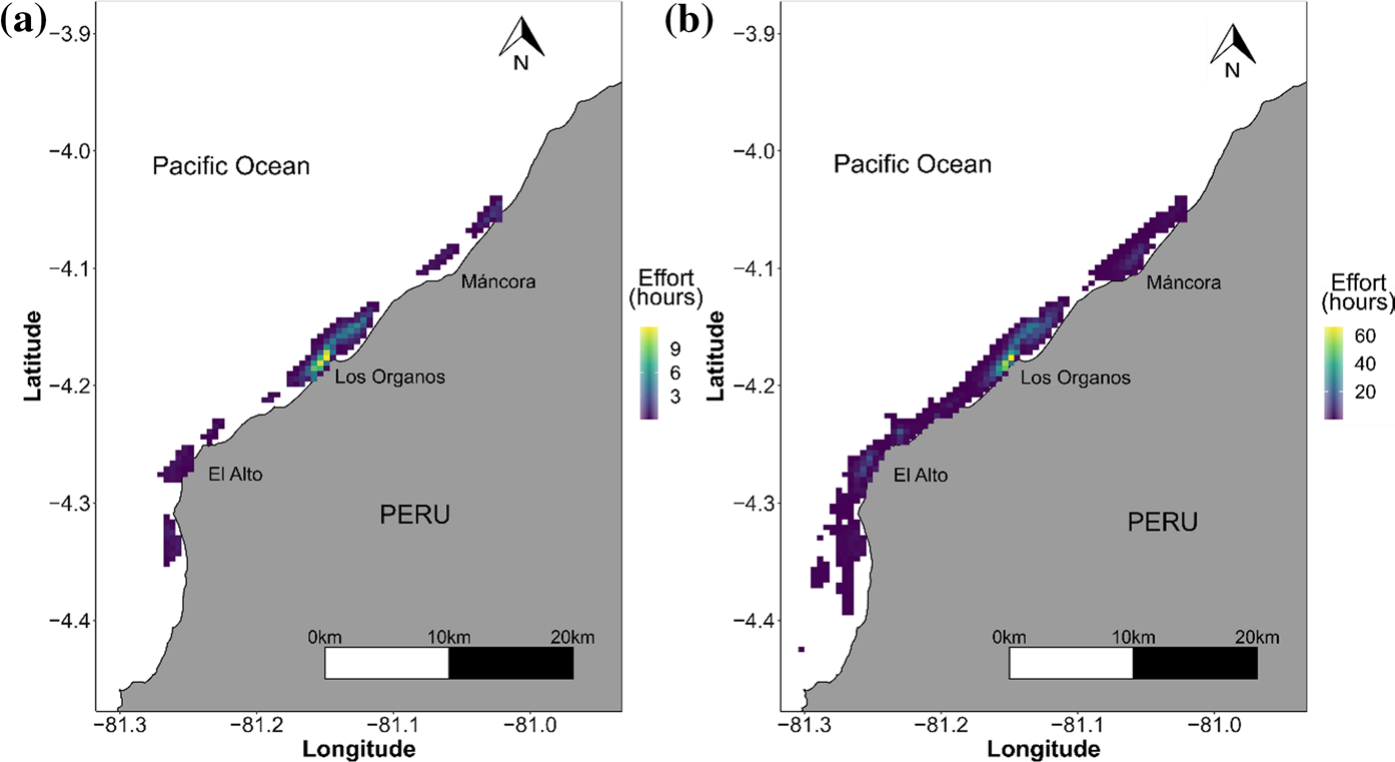
Spatial distribution of trawling activities (effort in hours trawling) from **a** data collected by observers, **b** fishers self-reporting via App from October 2019 to March 2020

**Fig. 3:**
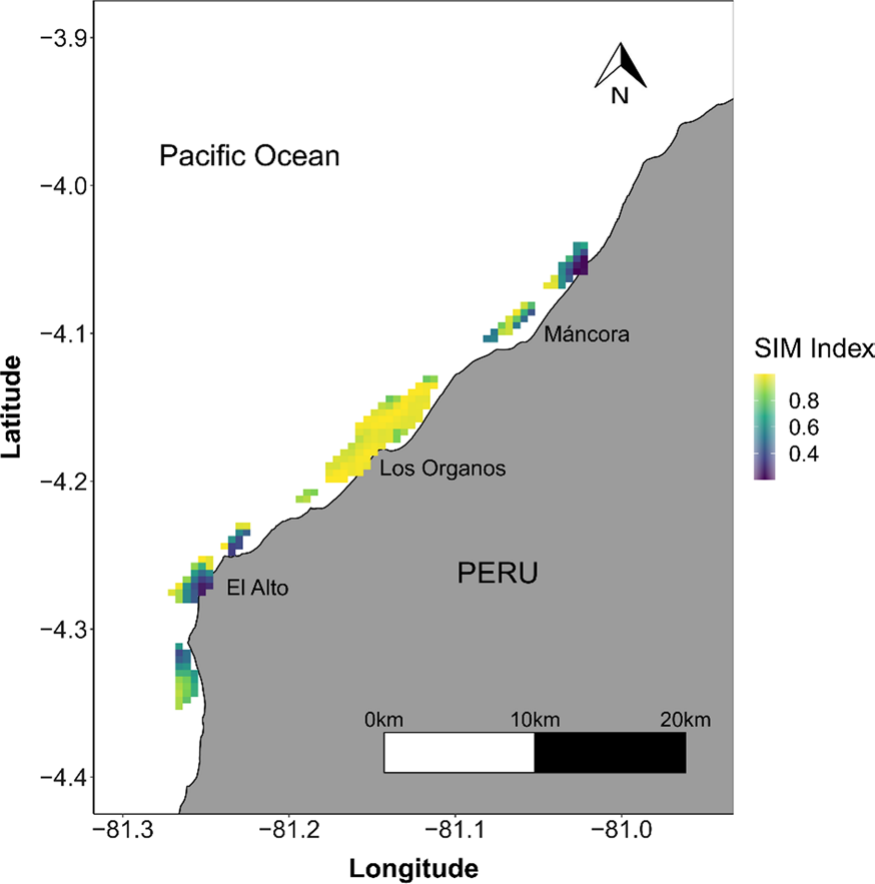
Map comparison between fishing effort in small-scale shrimp trawl vessels reported by observers and fishers using the Similarity of Means Index

Spatial patterns of fishing effort (time spent trawling) were very similar between data reported by observers and fishers (Fig. 2). Most trawling activities occurred in the proximity of the fishing town “Los Órganos”. The SIM Index was very high across months (minimum value in December 0.62 and maximum in March—0.90, Supplementary material 3, table S1), with an overall index of 0.815 for the 6-month study period. The greatest similarity in spatial patterns occurred in the main fishing area located in front of the fishing town Los Órganos, while in smaller, less frequently fished areas, the spatial patterns were more dissimilar (Fig. 3, e.g. north of the study area where observers recorded relatively higher levels of effort). These differences could be due to the relatively smaller number of trips conducted in these areas by fishers and observers (Supplementary material 3, Fig. S3). While the spatial footprint was very similar, a greater spatial footprint was revealed by fishers, due to the greater amount of data available from their submissions via the App.

### Temporal changes in discards

**Fig. 4:**
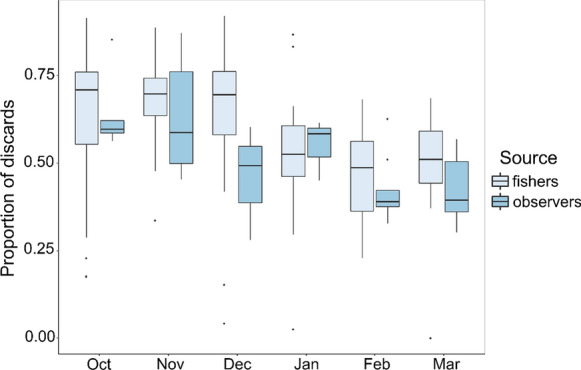
Proportion of discards reported by fishers and observers in small-scale shrimp trawl fisheries from October 2019 to March 2020 in northern Peru. Interquartile range (boxes), median (bold lines), 95% CI (bars), outliers (points)

There was no significant difference in the proportion of discards reported by fishers and observers (F = 2.47, df = 1230, *p* = 0.11). The main discarded groups were algae, fish and crabs and a discussion in temporal trends is available (Mendo et al 2020). Trends in the proportion of discards over time were consistent between observers and fishers and showed significant variations between months (F = 10.18, df = 5230, *p* < 0.001). The mean proportion of discards was higher Oct-Dec 2019 and significantly lower from Jan–Mar 2020 (Fig. 4). In December, there was a discrepancy in the proportion of discards reported between fishers and observers, with fishers reporting about 20% more discards than observers. This might be due to the small number of trips conducted by observers in December (n = 3), which may not have been a representative sample of fleet activity.

### Spatial patterns in proportion of discards

**Fig. 5:**
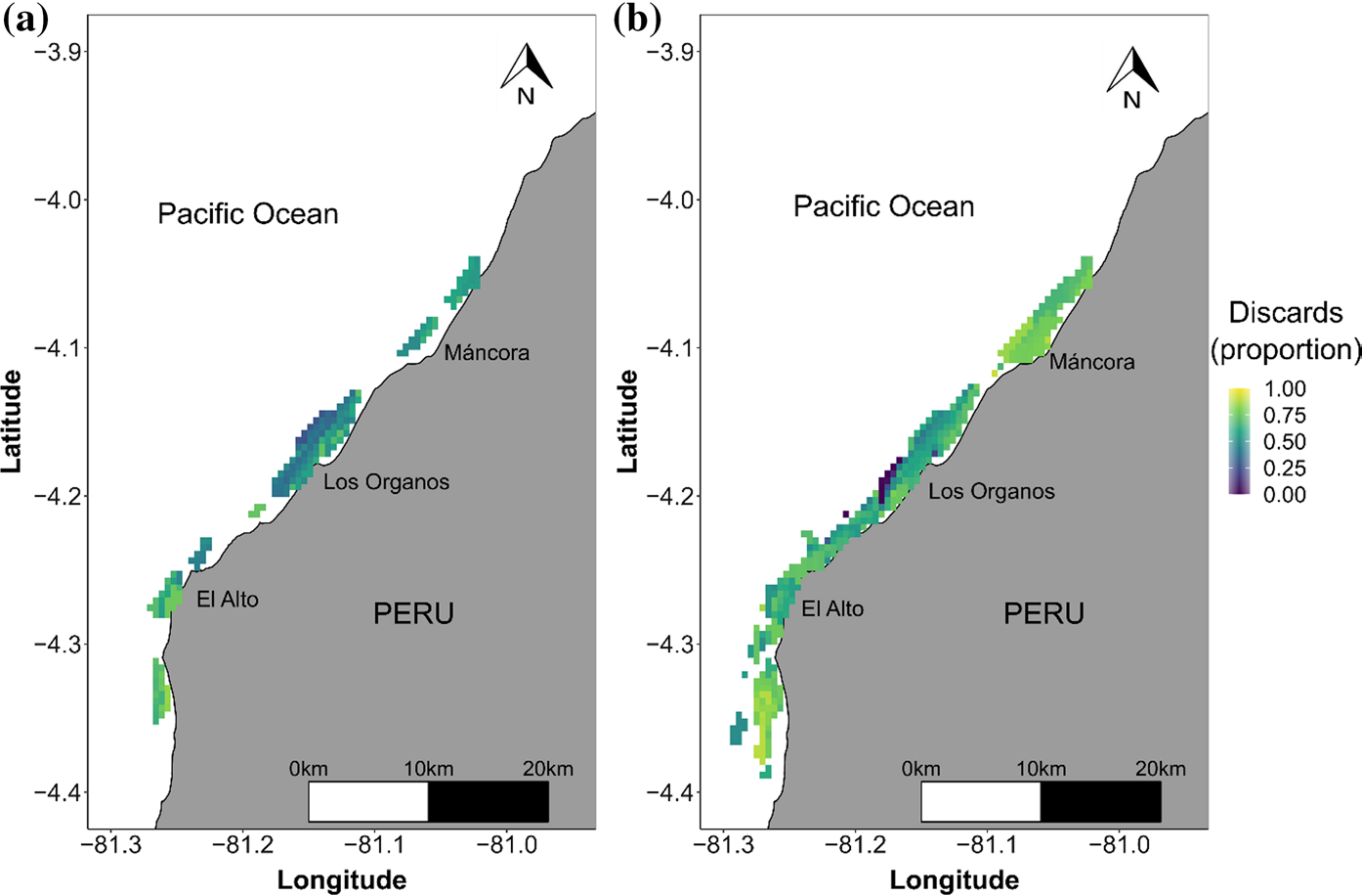
Spatial distribution of discards (as a proportion of total catch) from **a** data collected by observers, **b** fishers self-reporting via App in small-scale shrimp trawl fisheries from October 2019 to March 2020 in northern Peru

Spatial patterns of discards were very similar between data reported by observers and fishers (Fig. 5). Overall, the highest proportion of discards were reported south of the study area. In the main fishing area around Los Órganos, a reduction in the proportion of catch discarded was observed at greater distances from the coast (Fig. 5). The SIM Index was very high across months (0.87–0.98, Supplementary material 4, Table S2), with an overall index of 0.96 for the study period, which shows that there was broad agreement between the areas where fishers and observers both recorded lower or higher proportions of discards. A greater coverage on the spatial pattern of discards was achieved with fishers’ submissions.

## Discussion

The present study demonstrates that low-cost, fisher-led reporting technologies such as mobile Apps can be used successfully to improve the assessment of the impact of shrimp trawl small-scale fisheries. Finely resolved spatio-temporal data on fishing effort (time spent trawling) and discards were available for the first time from a fishery in northern Peru operating illegally within inshore areas (5NM). Self-reporting by fishers identified the same areas subjected to high fishing pressure and similar spatio-temporal trends in discards as data collected by on-board observers. Moreover, use of self-reporting allowed far greater spatial coverage than was possible with the lower numbers of observer trips, as is usually the case for SSF, where on-board observer programs are rare. In fact, in Peru, there is no on-board observer program for any SSF. Given the very limited information on trawling activities and discards in small-scale fisheries worldwide (Pérez Roda 2019; Suuronen and Gilman 2020), this represents a useful approach to collecting reliable fisheries data.

About a quarter of fishers’ submissions with spatial information did not provide associated catch (total weight and weight of main commercial species) data. This was observed for eight fishers and seemed to happen at random during the study period. This shows that even though fishers used the tracking feature in the App, they sometimes neglected to add complementary catch information. There were high inter-individual variations in reporting practices, with some fishers reporting quite often and others less frequently. It would be useful in the future to use behavioural sciences to understand what drives these motivations (e.g. Clary and Snyder 1999) and how to increase fisher participation. For 37 trips, discard data were available with no associated spatial data. This could be either because fishers neglected to start tracking or stopped the tracking function in the App.

It is widely recognised that self-reporting offers an opportunity to cover a larger proportion of the fleet with lower costs compared to observer programmes (Starr 2010). Moreover, high quality data, comparable to those collected by observer sampling has been achieved in several fisheries (Starr and Vignaux 1997; Hoare et al. 2011; Mion et al. 2015; Campbell et al. 2021; Marshall et al. 2021; Tilley et al. 2020). However, self-reporting has also been criticised; specifically, the lack of time, motivation and training of fishers which may lead to inaccurate reporting (Lordan et al. 2011; Sampson 2011; Mangi et al. 2015). We addressed these constraints by adhering to principles of innovations that increase their rate of adoption, such as relative advantage, complexity, and trialability (Rogers 2003). The relative advantage is the degree to which an innovation is perceived as advantageous. The motivation of fishers was clear as collectively they had already agreed that finding ways to reduce bycatch and discards and to adopt more sustainable fishing practices was potentially a way to legitimise the fishery. A more tangible advantage was obtaining a cell phone and associated monthly data costs. Several self-reporting trials have used incentives to increase fisher participation, for example, allowing fishers to access cod fishing grounds they would not have been otherwise been able to access in Germany (BLE 2018), reducing bycatch in order to maximise yields in the US scallop fishery (O’Keefe and DeCelles 2013), or in the form of direct payment for reporting (Ticheler et al. 1998). Complexity refers to the difficulty of understanding and using the App. We developed a mobile application with a simple design where only essential data were required to be filled in by fishers. Trialability, which refers to the degree to which an innovation may be experimented on before a full trial, was followed by inviting fishers to comment on the design of the App and on early iterations of the functionality of the App. While not fully developing a process of co-design with users as suggested in Nthane et al. (2020), this process helped to engage fishers, and increased the likelihood that the App would accommodate the operational constraints of using the App whilst fishing.

Unfortunately, this project was of a short duration and affected by COVID-19 in March 2020. Therefore, evaluation of declines in reporting over extended periods (Lordan et al. 2011) was not possible. Data on changes in target species by fishers was also only collected anecdotally by observers and could have affected the trends in submissions per fishers over time. For example, increased catches of shrimp during the summer months (Jan-Mar) led to a decrease in the price, to the point where some fishers chose to change target species to force buyers to increase the price again. Likewise, the number of interventions (confiscating catch and gear) by the Peruvian Navy increased from January onwards, which prevented fishers from going out fishing (Gomez, pers. obs.).

Low-cost self-reporting approaches have the potential to improve or even initiate data collection programmes to monitor small-scale fisheries discards, which remain widely unstudied globally (Suuronen and Gilman 2020). However, it is important to recognise that the utility of these approaches is context specific and establishing the appropriate framework and conditions for self-reporting approaches to work successfully is more important than the underpinning technology. While we suspect that the levels of engagement were initially relatively high due to the high level of fishers’ support (in order to generate information on their impact as means to start discussions with government towards legitimisation of the fishery), we believe that constant review of their submission data and frequent communication with them might have improved fisher engagement in reporting. Communicating effectively, transparently, and consistently with fishers can help to build trust. Helping to facilitate collective understanding of the challenges and potential solutions can provide the necessary motivation for fishers to participate in self-reporting approaches. Subject to achieving these conditions, combining spatial data with catch data can provide a powerful tool to identify fishing grounds, areas of high discard risk and other important information for fisheries management and conservation. These approaches also provide an opportunity to represent SSF activities in the context of competing demands on marine resources and spatial management related to the blue growth agenda (Cohen et al. 2019).

## Supplementary Information

Below is the link to the electronic supplementary material.

Supplementary file1 (DOCX 133 kb)

## Data Availability

The data presented is not available as it involves sensitive information on individual fishing activities. It was made clear to participants that data was going to be presented in aggregated form as presented in the publication.

## Code availability

The mobile application Pescar App (McCann and Mendo 2019), which runs on devices using the Android operating system was developed openly on GitHub under the terms of the open-source MIT License. https://zenodo.org/record/3519231#.X7OpOlDgqUk.
