# Supplementary material for: Assessing discards in an illegal small-scale fishery using fisher-led reporting
Source: Rev Fish Biol Fish. 2022 Mar 28;32(3):963–74. doi: 10.1007/s11160-022-09708-9 (PMC8958935; doi:10.1007/s11160-022-09708-9)
Supplement: Supplementary file 1 — Supplementary file1 (DOCX 133 kb) [file 11160_2022_9708_MOESM1_ESM.docx]

**Supplementary Material 1. Pescar App user interface**


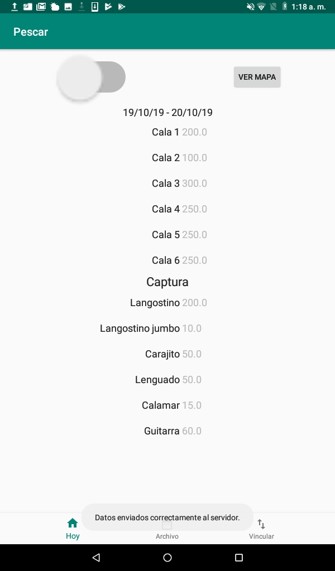

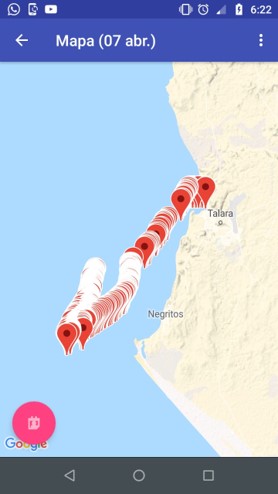


Fig. S1. Pescar App showing a) the user interface with a button on the upper left hand side to start tracking the trip and the fields that fishers had to complete; b) an example of a fishing trip being tracked that fishers could access in real time

Supplementary material 2


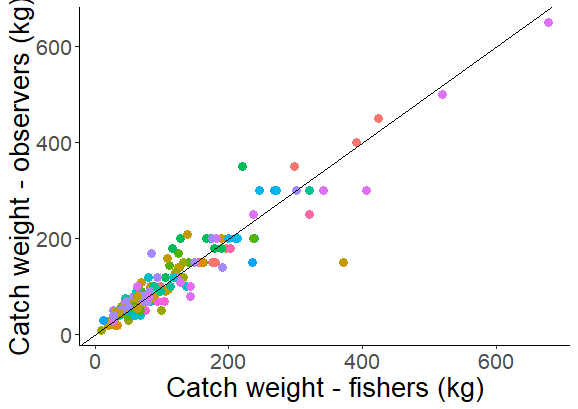


Fig. S2. Comparison between weight estimated by fishers and weight reported by observers using scales. Each colour denotes a different skipper (from 12 different vessels). Intercept=-1.14, slope=0.98, R2=0.898

Supplementary material 3

Table S1. Comparison between maps of fishing effort produced by observers and fishers through the Similarity in Means Index from October 2019 to March 2020

| Month | SIM Index (mean, variance) |
| --- | --- |
| October 2019 | 0.81, 0.05 |
| November 2019 | 0.71, 0.09 |
| December 2019 | 0.62, 0.12 |
| January 2020 | 0.85, 0.02 |
| February 2020 | 0.79, 0.03 |
| March 2020 | 0.90, 0.01 |


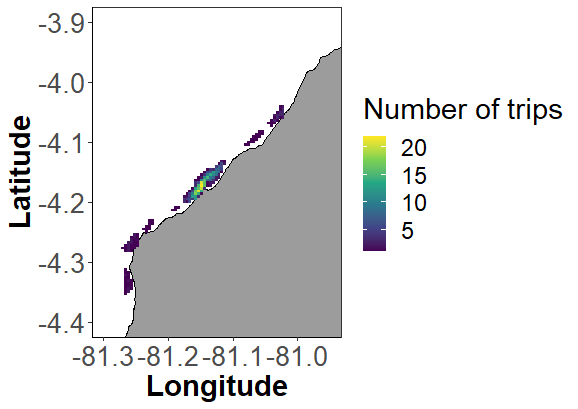

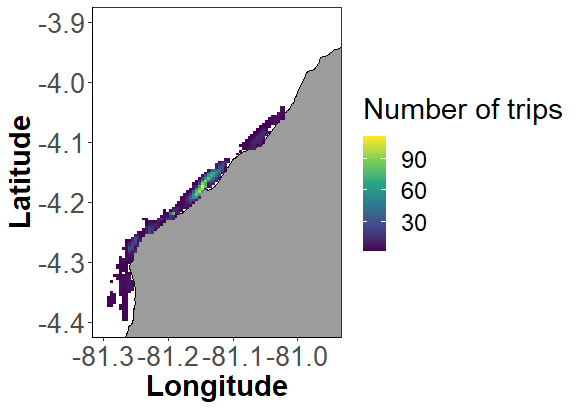


Fig. S3. Number or trips conducted in each grid cell by a) observers and b) fishers from October 2019 to March 2020

Supplementary material 4

Table S2. Comparison between maps of discards produced by observers and fishers through the Similarity in Means Index from October 2019 to March 2020

| Month | SIM Index (mean, variance) |
| --- | --- |
| October 2019 | 0.98, 0.0005 |
| November 2019 | 0.96, 0.001 |
| December 2019 | 0.87, 0.07 |
| January 2020 | 0.97, 0.0005 |
| February 2020 | 0.98, 0.004 |
| March 2020 | 0.95, 0.003 |


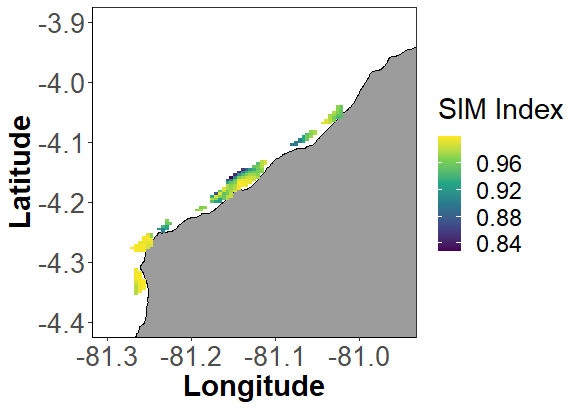


Fig. S4. Map comparison between discard proportions reported by observers and fishers using the Similarity of Means Index.
